# Catechin ameliorates depressive symptoms in Sprague Dawley rats subjected to chronic unpredictable mild stress by decreasing oxidative stress

**DOI:** 10.3892/br.2020.1326

**Published:** 2020-07-07

**Authors:** Amita Rai, Meghna Gill, Manas Kinra, Shetty Raghavendra, Nandakumar Krishnadas, C. Mallikarjuna Rao, Suhani Sumalatha, Nitesh Kumar

Biomed Rep 11:79-84, 2019; DOI: 10.3892/br.2019.1226

Subsequently to the publication of the above article, the authors have realized that the text contained certain errors that were not corrected prior to publication. First, in the Materials and methods section on p. 81, left-hand column, in the subsection ‘*Assay for antioxidant catalase levels*’, on the fourth line, the range of OD values should have been presented as **0.3-0.5**, not as 3-5. Secondly, on pp. 82 and 83, in the figure legends for Figs. 1-3, the text should have been written to state: ‘Rats were subjected to CUMS and treated with escitalopram or **catechin for** 8 weeks.’ (i.e. not ‘catechol from’). Finally, also in the legend for Fig. 3, the subsequent (i.e, third) sentence should have been written as: ‘After 8 weeks animals were sacrificed and the brains were collected for measurement regarding antioxidant levels; **catalase** and SOD activity, as well as GSH and MDA levels were determined.’

The authors regret that these errors were allowed to enter into their text, and were not corrected prior to publication. The authors also apologize to the readership for any inconvenience caused.

